# Lambs' temporal bone anatomy under didactic aspects

**DOI:** 10.1590/S1808-86942011000100009

**Published:** 2015-10-19

**Authors:** André Gurr, Marc David Pearson, Dazert S

**Affiliations:** 1 Dr. med. (ENT-Specialist); 2 Resident; 3 Prof. Dr. med. (Director of the Dept. of Otorhinolaryngology, Head- & Necksurgery, Ruhr-University Bochum, Germany) Dept. of Otorhinolaryngology, Head & Necksurgery, Ruhr-University Bochum, Germany

**Keywords:** anatomy, middle, ear, temporal bone, sheep, veterinary

## Abstract

Human temporal bones in teaching ear surgery are rare. The lamb's temporal bone might be a possible alternative.

**Material and Methods:** Temporal bones of the lamb were dissected with a typical temporal bone lab drilling program. We included a mastoidectomy, endaural approaches, but also analyzed the outer appearance, the external ear canal and the hypotympanon. Some steps differed from preparation done in humans. The morphometric results were compared to the known anatomy of human in order to verify the lambs' temporal bone for suitability in otosurgic training.

**Results:** The lambs' temporal bone appears smaller than the human one. We found a bullous extended hypotympanon located under the external ear canal. The tympanic membrane is very similar to the human one. The external ear canal is smaller and shorter. The ossicular chain shows analogies to human one.

**Discussion:** This study shows, that especially the middle ear, the tympanic membrane and the external ear canal are morphologically equal to the structures found in human temporal bones. The lamb seems feasible for teaching the anatomy of the ear. The smaller scales of some structures, especially the outer components of the temporal bone are a disadvantage.

**Conclusions:** The lamb seems to be an alternative in teaching ear surgery.

## INTRODUCTION

The use of human temporal bones is limited by their bad availability. The usual alternatives for teaching ear surgery are rare and consist of virtual temporal bones or models. Unfortunately they are not suitable for training chain reconstruction or preparatory work on the middle ear. Temporal bones retrieved from animals might help to reduce the need for human biologics^1^, ^2^, ^3^, ^4^, ^5^, ^6^, ^7^, ^8^ in teaching and research. In order to verify the feasibility of the lambs temporal bone for ENT-education we designed this morphometric study.

## MATERIAL AND METHODS

### Prearrangement

We retrieved 12 heads of lambs, aged between 8 to 11 months from a local slaughterhouse. No animal was killed for research purposes. The skin was removed, a sagittal separation of the heads was done and ventral parts of the heads were removed as well as the the brain. These probes were fixated in formaline solution of 3 % for 6 to 8 weeks. The appending soft tissue was removed from the fixated temporal bone in order to expose the mastoidal plane and the external ear canal.

### Preparation

Before any manipulation on the temporal bones we examined and described the outer anatomy. As a first dissection step the bullous hypotymanon was opened. This allowed a good view from inferior into the middle ear.

Based on this approach we opened the external ear canal, leaving the fibrous anulus of the tympanic membrane untouched in order to get a view on the tympanic membrane and the external ear canal suitable for morphometric analysis.

The mastoidectomy was performed analogous to the one in humans, between the temporal line and the posterior wall of the external ear canal including the whole mastoidal plane. This procedure was done until the semicircular canals, the access to the middle ear, the facial nerve and the incus were visible.

As last step the ossicular chain was removed. This made morphometric studies on the promontory possible.

### Measurement

The identified structures in all anatomical preparation were measured by an optical measurement procedure. The optical views were grabbed by a digital single lens reflex camera which had been adapted by a C-Mount to the microscope or a 0° degree endoscope. The light source was either the microscope itself or a standard surgical light fountain used in endoscopic surgery. We laid a small strip of foil containing units of 1 mm into the field of view. This strip was used as a reference for measurement calibration. The calibrating strip was positioned as near as possible to the measured structure.

The gauges were done with the help of the D-Cell Image analysis system which is a two-dimensional, pixel orientated software. After calibration of each single shot the anatomical parameters were acquired. It was imported to use views nearly perpendicular to the measured structure in order to avoid faulty results.

We determined the length and the smallest diameter as well as the diameter at the orifice of the external ear canal. The angles in two directions of the external meatus were measured. The tympanic membrane was gauged in length and height, as well as its nook to the external ear canal. In the middle ear spaces we quantified the length and sizes of all ossicules first in the middle ear and again isolated after chain removal in order to proof the measuring method.

On the promontory, the sizes of the oval and the round window and the distance of this structures were measured as well. The position of the cochlea was determined and described, but not quantified.

The structures found in mastoidectomy were described and partially measured. The acquired data was especially the angle of the short incus process to the lateral semicircular canal.

## RESULTS

### Outer appearance

Macroscopically the lamb's temporal bone appears smaller compared to human with a lot of similarities. The external ear canal is positioned humanlike and the mastoidal plane seems to have the same structural morphology.

The distance between temporal line and cranium was 1,71 cm on average. The atlanto-occipital joint is vectored backwards as well as the foramen magnum. A styloid process cannot be found. The mastoid strongly expands inferiorly.

**Figure 1:**
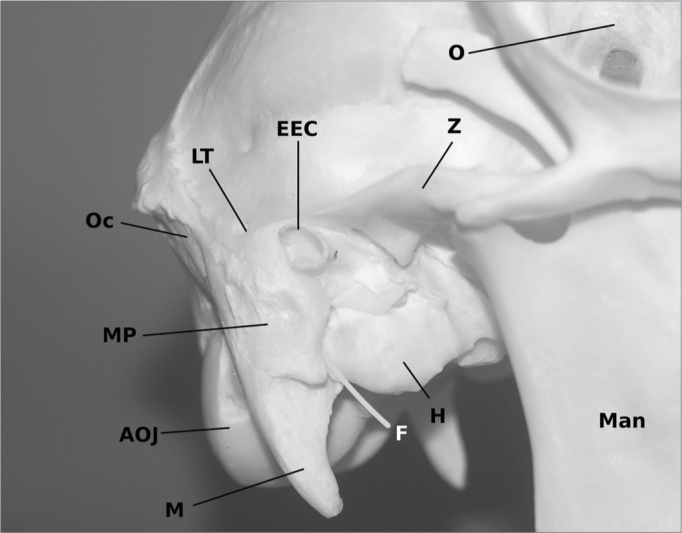
The skull of a sheep with view on the right ear. The human like external ear canal (EEC) is cranially separated from the skull by the zygoma (Z) which expand to the linea temporalis (LT). Below the big hypotympanal bulla (H) is visible. The mastoid plane (MP) and the mastoid process (M) can also be identified. The course of the facial nerve (F) is marked yellow.Man = Mandibula, O = Orbita, AOJ = Atlanto-occiptal joint, Oc = Occiput.

We found a bullous structure consisting of a thin bony layer, later identified as the hypotympanon, which measured 11,4 mm in depth and 23,2 mm in length averaged (Figure 1).

External ear canal and tympanic membrane

**Figure 2:**
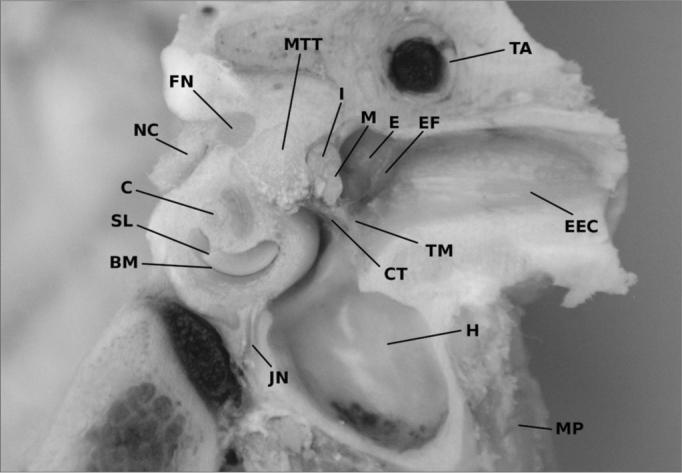
A frozen section of a lambs' temporal bone (left ear) showing the middle ear, the external ear canal (EEC) and the inner ear as a front view. The cochleas' turns (C) are well visible with the osseus spiral lamina (SL) and the basilar membrane (BM) inside. Above the tensor tympani muscle (MTT) can be identified on its way to the malleus handle. The cochlear nerve (CN) is to be identified within the internal ear canal with anatomic relation to the facial nerve (FN). The external ear is opened cutting the tympanic membrane (TM) and the epitympanic fold (EF). In the middle ear spaces the body of the incus (I) and parts of the malleus (M) can be seen. Caudal the big hypotympanal bulla (H) is prominent. MP = Mastoidal plane, TA = temporal artery, E = epitympanon, JN = Jacobsons' nerve

The bony external ear canal is orientated on the same axis as in humans but it has smaller scales. Its length was 12,6 mm in the mean with a maximum of 15,7 mm and a minimum of 12,4 mm. The canals diameter was 2,9 mm on average (Figure 2).

The tympanic membrane appears circular as it does in humans. The epitympanon shows, differing from humans, a long extension with an averaged length of 4,1 mm. The size of the tympanic membrane was 9 mm in diameter.

### The middle ear

**Figure 3:**
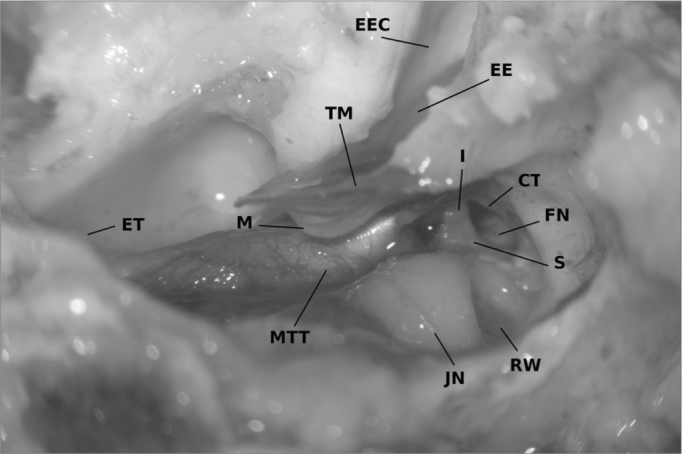
Lambs' middle ear anatomy (right ear) seen from the opened hypotympanon. The external ear canal (EEC) has been opened for measurements and exposes the epitympanal extend (EE) of the tympanic membrane. The tympanic membrane (TM) with the curved malleus' handle (M) can be seen. The tensor tympani muscle (MTT) inserts directly on the handle with the chorda tympani (CT) bending around. The stapes (S) is encircled by the facial nerve (FN) and connected to the long process of the incus (I). RW = round window, JN = Jacobsons' nerve, ET = Opening to the Eustachiian tube

The hypotympanal opening provides a good overview of the middle ear cavity. Malleus, incus and stapes are visible, as well as chorda tympani and facial nerve. The malleus is structured like the human one. The handle is 5,6 mm long and has a clearly curved shape. Connected to it, the tensor tympani muscle can be found. The chorda tympani bends around the tensor tympani chord (Figure 3).

The malleus head is connected to the incus. This shows two processes perpendicular to each other. The so called long process in humans, has an average length of 1,3 mm in the lamb. The other one measures 2,6 mm in mean. The incus body appears to be very similar to the human one. The stapes head, branches and footplate are comparable to the ones found in humans as well. The head measures 0,68 mm in diameter on average with the following branches of 1,6 mm in length. The footplate is 2,1 mm long and has an average width of 1,1 mm.

### The mastoidectomy

**Figure 4:**
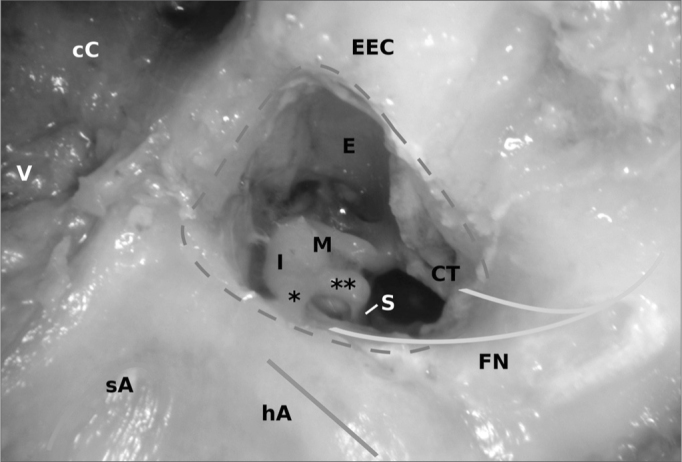
A mastoidectal approach to a lambs' middle ear (right ear). The bony labyrinth block is enclosed by cranial cells (cC) containing fat and a spongiform structured skull occipital and caudal. The axis of the lateral (blue) and the superior (green)  semicircular canal are made visible. The access to middle ear is artificial by a partial resection of the external ear canal. The stitched grey line shows the margin of this opening. The facial nerve (FN) encircles the stapes (S) as in humans and serves the chorda tympani (CT). The incus (I) is to be found medial of the lateral arcade. M = Malleus, E = Epitympanon, V = venous vessel, * = short process, ** = long process.

The linea temporalis as well as the spina suprameatum can easily be identified and anatomically directly followed by the mastoidal plane. The lambs mastoid shows no pneumatization in the lamb. The cranial cells are filled with fat and venous vessels. The preparation is difficult. The inner ear structures are enclosed in a compact bony block surrounded by spongiosa. With the absence of aerated cells no antrum can be identified. In order to see the lamb's middle ear anatomy from the mastoid the external ear canal has to be opened. The incus body can be found medial of the lateral semicircular canal which differs from human anatomy (Figure 4). As in humans the posterior semicircular canal can be found perpendicular to the lateral one pointing upwards. A sigmoid sinus cannot be found. Cranially the mastoid has no anatomical relation to the middle cranial fossa. Instead one can find a bony lamella, which is only covered by the animals skin, muscle and outer ear. Medially and cranially the dura of the young sheep can be identified.

The dissectional work in the mastoid is difficult, caused by some variations and the missing pneumatization as well as the missing antrum. The inner ear structures are difficult to identify, because of the late change of color in the bone. Additionally the semicircular canals seem to be smaller. The facial nerve and its anatomical relation to the lateral semicircular canal is similar to human anatomy.

## DISCUSSION

The existing studies of animal's temporal bone anatomy rarely focus on the didactic use of those bones. Most measurements are done using computer tomography^1,^^7^ as well as with anatomical dissection^1,^^2,^^6^.The surgical orientated dissection is rare.

### Outer morphology

The distance between cranium and zygoma is 1,6 cm on average. In humans these two anatomical structures can be found on one identical plane only separated by the linea temporalis. A fact that has not been described by other authors before, presumably because they used complete heads in a CT-scan^7^ or focused especially to the middle ear. Another special formation is the big bullous hypotympanon. Its function is unclear. We assumed that the missing pneumatization of the mastoid might be balanced weight-wise by a large aerated hypotympanon. The easy access to the middle ear via the hypotympanon might prove to be an additional advantage for teaching and understanding middle ear anatomy.

Other than in humans the atlanto-occipital joint is vectored backwards. A fact that has been reported before from the pig^3^. This can be expected at all quadruped animals. The mastoidal plane is similar to human one even though it is smaller. It has a prominent process, which reminds of a strong human styloid process. The facial nerve extrudes the skull in close anatomical relation. It might be discussed whether in sheep the styloid process and mastoid are combined in just one single structure.

Opening the mastoidal plane can be taught easily, as these bones have the typical landmarks needed^8^, but limitations will be found in the mastoidectal procedure.

### External ear canal and tympanic membrane

The tympanic membrane of the lamb is as round as it can be found in humans except for an epitympanal extension of approximately 4 mm. The average diameter of the tympanic membrane is nearly the same as in humans with a scaling factor of 0,93. The position and the angle of the external ear canal are the same too, but the bony portion is very small compared to human one. With approximately 3 mm it is even to narrow to use common microsurgical instruments^8^. Endaural use without a modification is not possible. An opening of the caudal external ear without removal of bone directly surrounding the tympanic membranes can give better access but reduces realism compared to human biologics.

The tympanic membrane has an expanded epitympanic fold. Opening of this extension provides a direct view on the ossicular chain. In humans, parts of the posterior ear canal wall have to be removed to see major parts of the ossicular chain. On one hand this can be seen as a disadvantage as the missing removal of bone can not be trained, but on the other hand it is an easy way to understand the position of the ossicular chain, the facial nerve and the tympanic chord in relation to the tympanic membrane.

In lamb the tympanomeatal angle is very small and the anterior ear canal is only 1 to 2 mm away from the tympanic membrane. This should be considered when resecting parts of the external ear canal. Under didactic aspects this is a good training for learning the use of a drill.

### The middle ear

Middle ear structures in the lamb do not differ much from human ones. The handle of the malleus has the same angle and position. Exactly as in humans the tensor tympani muscle inserts at the proximal part of the manubrium.

The incus is also very similar. The angles of the processes differ from the human ones. The so called long process in humans is short in the lamb which might lead to problems when fixing a piston prosthesis. The stapes morphology is also similar to the human one. We can find a stapes head, branches and a footplate. A successful use for training surgery of otosclerosis has been described using adult animals^5^.

**Figure 5:**
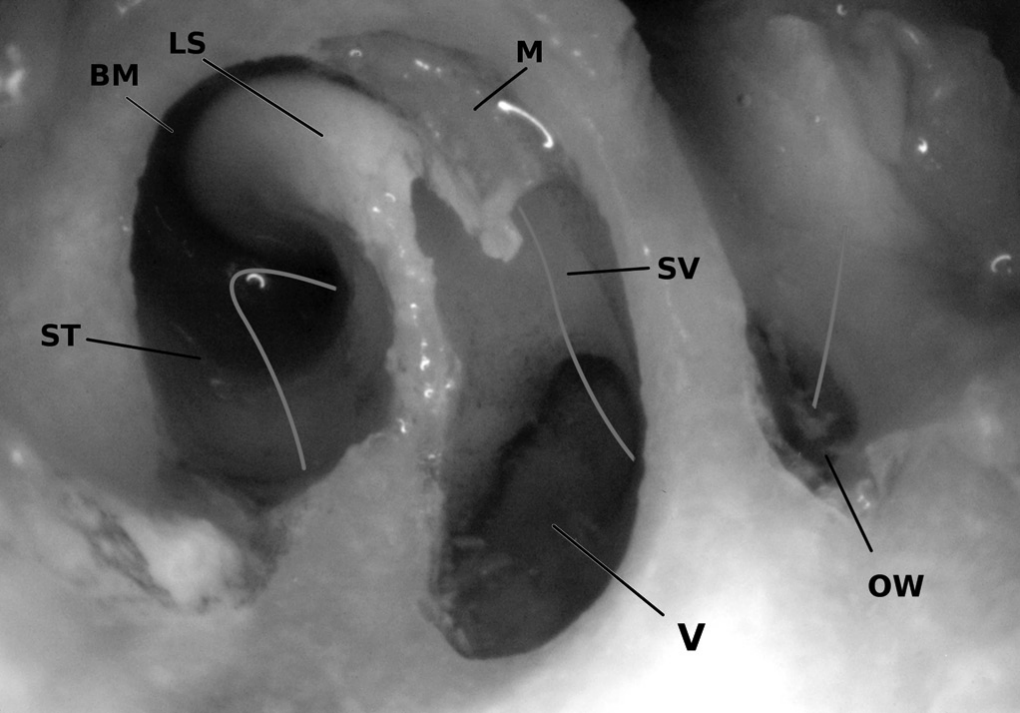
An opening of the lambs' round window (left ear). The stapes has been removed to inspect the oval window (OW). Cranial remains of the covering membrane (M) can be seen. The osseus spiral lamina (SL) has partially been resected to give way into the Scala vestibuli (SV) and the vestibule (V). The scala tympani (ST) as well as the basilar membrane (BM) can also be seen. The green line clarifies the way of sounds' energy. After entering via the oval window it passes through the vestibule to the Scala vestibuli up to the cochleas' tip and continues within the Scala tympani and then is emitted via the round windows' membrane.

The promontory formation in lambs and humans is similar as well. We could find the same positioning with only slightly different scales and distances. Exercises like tympanoscopies with manipulation of the round window are imaginable if an endaural approach is performed. This implies using the external ear canal after partially resecting it as described above. In literature smaller structures within middle-, and inner ear have been described with a scaling factor of 2/[Bibr bib3][Bibr bib2] ^7^. It is doubtful whether these biologics are feasible for training cochlea implantations. The standard electrodes, constructed for the use in humans are too long and wide. Teaching cochleostomies or an opening of the round window is possible using the lamb. Opening the round window gives a great view onto the anatomic structures within (Figure 5) and helps to understand the principle of sound conduction.

### Mastoidectomy

The mastoid is not pneumatized, which reduces its use as a training model for a classic mastoidectomy. We noticed large cells filled with fat and venous vessels, that were difficult to expose.In ct-scan based studies other authors also report of a fatty filling of the superior lambs mastoid^2,^^3^.After removal of the fat, the bony block of the labyrinth is free.

The drilling work on the labyrinth is difficult as well. The position of the lateral semicircular canal varies slightly from the human one. For better orientation the posterior wall of the external ear canal should be identified and resected until the incus can be seen. Mostly this ossicle can be found medially of the lateral semicircular canal and not laterally as in humans. Different from humans, the bony structures near the inner ear formations are not colored yellow nearby, which makes the dissectional work more difficult. The change in color only occurs very deep in the labyrinth block.

The fact that the dura is missing towards the intercranium is not necessarily a disadvantage for educational purposes, because the thin bony layer between zygoma and cranium resembles human anatomy. The sigmoid sinus as a very important landmark cannot be seen^8^.

The higher position of the incus in relation to the lateral semicircular canal within the antrum and the short process pointing towards the semicircular canal are important landmarks in mastoidectomy and for posterior tympanotomy. The preparation of the chorda-facial angle offers a view on the top of the incus. The promontory with its ideal position for cochleostomy can be reached with some additional drilling.

Therefore the mastoid of a lamb can not be recommended for beginners to teach anatomy but might be usable for training the handling of drill and suction. These temporal bones seem to be a good addition in the learning process for advanced learners as they should be able to solve problems also in anatomic variation. If the lamb's varied morphology of the mastoid is accepted by learner and teacher it can be used to train chain and interposition work from a posterior approach, especially as the morphology of chain, promontory, facial nerve and lateral semicircular canal are similar to a high extent. Especially the facial nerve's anatomy can be taught ideally.

### General view

Temporal bones of the lamb have an external ear canal, chain and inner ear structures similar to humans. Unfortunately some scales differ if compared to humans. Most sizes are smaller, which is a challenge for trainees. As a lot of morphology is similar, these bones might be used for teaching anatomy and surgical techniques as others described before^2,^^5,^^7^. The most common alternative to human temporal bones are virtual trainers based on CT-scans of human temporal bones^9,^^10^. They are more appropriate than animal models for teaching especially the mastoids anatomy, but they are not useful for teaching the coordination of drill, suction and other microsurgical tools. These surgical skills can be improved by using animal biologics. Digital trainers are also not applicable for work on the ossicular chain, as the insertion of prostheses is impossible. This disadvantage does not exist in animals. Reconstructive steps can be taught ideally. Until today virtual models do not offer color information like animal biologics, but the first studies for full colored datasets exist^11,^^12^ and will advance digital techniques in future.

A combination of models, virtual trainers and animal temporal bones will be the best choice for teaching ear surgery, assuming difficulties in obtaining human temporal bones. Without doubt human biologics remain the best option today and in future.

Beside didactic purposes the easy access through the hypotympanon predestines the lamb as well as the adult sheep for studies on middle ear mechanics, implant movements, or implantable hearing aids on cadavers.

Because of its similarities to humans the lambs ear is ideal for electrophysiologic experiments^13^ as well as for experimental surgery^14^. The easy access to the middle ear allows implantation of materials. Recent studies report of successful osseointegration of prothesis into the mandibule^15^ and the middle ear^16^ of sheeps.

As in Europe restrictions because of the bovine spongiform encephalitis (BSE) exist, we recommend not to use sheep older than 12 months in these countries for hygienic and judicial reasons for didactic purposes. In non-european countries no limits are given. It should not be a problem to do research or teach with these animals in these areas^17^. Alternatively the pig can be used as well^3,^ ^4^. The described use of non-human primates^6^ does not seem to be very helpful, as apes skulls are less available than human temporal bones.

## CONCLUSIONS

The lamb's external ear canal is shorter and more narrow than the human one and can only be used for teaching if it is opened. The bullous hypotympanon opens additional options for teaching middle ear anatomy as well as for research in middle ear mechanics.

The lambs mastoid is not pneumatized. Training mastoidectomies on lambs cannot be recommended for beginners due to the varied anatomy and a difficult preparation. Dissectional training for experienced learners is imaginable.

Especially the middle ear shows a lot of similarities compared to human ones. It might be a good addition for training reconstructive techniques on the ossicular chain.

A combination of different didactic methods for ear surgery, as artificial models, animal temporal bones and virtual trainers might be the best way to reduce the need of human temporal bones.
